# Structure, localization and histone binding properties of nuclear-associated nucleosome assembly protein from *Plasmodium falciparum*

**DOI:** 10.1186/1475-2875-9-90

**Published:** 2010-04-08

**Authors:** Jasmita Gill, Anuj Kumar, Manickam Yogavel, Hassan Belrhali, SK Jain, Melanie Rug, Monica Brown, Alexander G Maier, Amit Sharma

**Affiliations:** 1 Structural and Computational Biology Group, International Centre for Genetic Engineering and Biotechnology (ICGEB), Aruna Asaf Ali Road, New Delhi, 110067, India; 2 European Molecular Biology Laboratory (EMBL), 6 rue Jules Horowitz, BP 181, F-38042 Grenoble Cedex 9, France; 3 Department of Biotechnology, Jamia Hamdard, New Delhi 110062, India; 4 Malaria Functional Genomics Facility, The Walter and Eliza Hall Institute of Medical Research, 1G Royal Parade, Melbourne 3050, Australia; 5 Biochemistry Department, La Trobe University, Melbourne 3086, Australia

## Abstract

**Background:**

Nucleosome assembly proteins (NAPs) are histone chaperones that are crucial for the shuttling and incorporation of histones into nucleosomes. NAPs participate in the assembly and disassembly of nucleosomes thus contributing to chromatin structure organization. The human malaria parasite *Plasmodium falciparum *contains two nucleosome assembly proteins termed PfNapL and PfNapS.

**Methods:**

Three-dimensional crystal structure of PfNapS has been determined and analysed. Gene knockout and localization studies were also performed on PfNapS using transfection studies. Fluorescence spectroscopy was performed to identify histone-binding sites on PfNapS. Extensive sequence and structural comparisons were done with the crystal structures available for NAP/SET family of proteins.

**Results:**

Crystal structure of PfNapS shares structural similarity with previous structures from NAP/SET family. Failed attempts to knock-out the gene for PfNapS from malaria parasite suggest essentiality in the parasite. GFP-fused PfNapS fusion protein targeting indicates cellular localization of PfNapS in the parasite nucleus. Fluorescence spectroscopy data suggest that PfNapS interacts with core histones (tetramer, octamer, H3, H4, H2A and H2B) at a different site from its interaction with linker histone H1. This analysis illustrates two regions on the PfNapS dimer as the possible sites for histone recognition.

**Conclusions:**

This work presents a thorough analysis of the structural, functional and regulatory attributes of PfNapS from *P. falciparum *with respect to previously studied histone chaperones.

## Background

In eukaryotic cells, DNA is present in a highly compacted form called chromatin. The repeating unit of chromatin is the nucleosome, formed from two histone H2A-H2B dimers and one histone H3-H4 tetramer around which 147 bp of DNA are wrapped [1]. Chromatin is highly dynamic, a characteristic that is vital in regulating nuclear processes such as transcription and replication which require access to DNA. Processes that influence chromatin fluidity include post-translational modifications of histones, incorporation of histone variants, and histone exchange by dedicated histone chaperones [2,3]. Histone chaperones are proteins that regulate the interaction of histones with other proteins and DNA and also prevent the highly basic histones from forming inappropriate aggregates [2,3]. In addition to playing an important role in histone exchange during nuclear processes, histone chaperones function in nucleocytoplasmic shuttling of histones, in histone storage, in nucleosome assembly and they act as a link between chromatin remodeling factors and histones [2,3]. Many different histone chaperones have been identified, including nucleoplasmin, Asf1 (anti-silencing function 1), HIRA proteins (Histone regulator A), Spt6 (Transcription elongation factor), ACF (ATP utilizing chromatin assembly and remodelling factor), CAF1 (Chromatin assembly factor 1), and NAP1 (nucleosome assembly protein 1) [2,3].

Malaria is one of the most common infectious diseases and remains an enormous public health problem. Malaria is caused by protozoan parasites of the genus *Plasmodium*, and the most serious form of the disease is caused by *Plasmodium falciparum *[4]. Thus, it is important to understand the fundamental biological processes of *P. falciparum*, which may provide avenues for the identification of new protein targets for development of new anti-malarials. The human malaria parasite *P. falciparum *contains two nucleosome assembly proteins, orthologs of eukaryotic NAPs, which have been previously termed PfNapL and PfNapS [5-8]. It has been previously shown that both PfNapL and PfNapS are present in all erythrocytic stages of the parasite [5,6]. It has also been shown earlier that PfNapS forms complexes with both histone tetramer and octamer and is predominantly localized in the nucleus in the asexual and sexual stages of the parasite [5,6]. PfNapL and PfNapS do not interact with each other and unlike PfNapL, PfNapS is able to deposit histones onto DNA [6].

In the present study, the structural basis of nucleosome assembly activity in *P. falciparum *was addressed by determining and analysing the crystal structure of smaller NAP in the parasite called PfNapS. Data are presented on the attempted gene knockout of PfNapS, its cellular localization and its histone binding properties. Attempts to generate parasites lacking PfNapS gene were unsuccessful indicating the likely essential nature of this gene for parasite survival. Using GFP-fused PfNapS deletion constructs, it is shown that PfNapS is most likely resident in the parasite nucleus. Fluorescence spectroscopy data indicate differential modes of PfNapS interaction with core histones versus the linker histone. Finally, a thorough comparative analysis of PfNapS is presented with structures of NAP/SET family proteins which include - PfNapL, yeast NAP-1, yeast NAP Vps75 (vacuolar protein sorting 75) and the functional domain of human SET/TAF-1b(β)/INHAT [7-12]. These studies provide basis for further exploration of nucleosome assembly as a relevant new target for development of anti-malarials.

## Methods

### Protein crystallization and data collection

Full length purified PfNapS protein (aa 1-269, molecular weight ~32 kDa) failed to crystallize and thus a shorter construct of PfNapS was produced containing residues 29-221. PfNapS seleno-met crystals were then successfully obtained at 20°C by hanging drop vapour diffusion [13] using l μl of 3 mg/ml PfNapS and l μl of 0.2 M di-ammonium tartrate with 20% PEG 3350 (mother liquor). A single crystal was soaked in a cryoprotectant containing higher concentration of mother liquor (30% PEG 3350 and 0.3 M di-ammonium tartrate) for 5 min and flash-frozen under a stream of nitrogen gas at 100 K. X-ray diffraction data were collected to 3.2 Å at BM14 beamline, ESRF, Grenoble. The seleno-met crystals of PfNapS belong to orthorhombic space group P2_1_2_1_2_1 _with cell dimensions of a = 96.18, b = 116.84, c = 138.65 Å having 3 dimers in the asymmetric unit (ASU). High-resolution native diffraction data to 2.8 Å resolution were collected at BM14 beamline, ESRF, Grenoble. These crystals also belong to orthorhombic space group P2_1_2_1_2_1 _with different cell dimensions of a = 95.79, b = 114.89, c = 139 Å. The diffraction images were processed and scaled with the HKL2000 suite [14].

### Phasing, structure determination, refinement and analysis

The structure of PfNapS was determined using selenium-SAD technique and phasing was achieved by utilizing a total of 24 selenomethonines (8 per dimer) in the ASU to 3.2 Å resolution using SHARP [15]. Only a partial initial model was built without side-chains using PHENIX [16] and this model was subsequently rebuilt manually using COOT [17]. The higher resolution structure was determined by molecular replacement technique using PfNapS seleno-met model. This model to 2.8 Å was refined using CNS ([18], Table 1). Final model was validated using PROCHECK [19]. All figures were generated using Chimera [20]. Least square fittings and structural alignment were carried out using LSQMAN [21]. Protein localization predictions were done using LOCtree [22].

**Table 1:** Data collection and refinement statistics

*Parameter*	*Native*	*Seleno-Met*
***Data collection***		

Space group	P2_1_2_1_2_1_	P2_1_2_1_2_1_

Cell Dimensions (Å)	a = 95.79, b = 114.89, c = 139	a = 96.18, b = 116.84, c = 138.65

Wavelength (Å)	1.0	1.0

Anomalous scatterer	-	Se

Resolution (Å)	50.0-2.8	50.0-3.2

Outer shell resolution (Å)^a^	2.9-2.8	3.31-3.2

No. of unique reflections	37166 (3101)	24009 (1781)

Redundancy	5.9	5.8

R_merge_^b^	0.68 (0.295)	0.142 (0.653)

Completeness (%)	100 (96.6)	99.7 (69.6)

**I/σ**I	23.5 (3.1)	10.16 (1.2)

***Refinement***		
	
Resolution range (Å)	50-3.0	
	
No. of reflections (test set)	35024 (3501) 0	
	
R_factor_/R_free _(%)	28.5/31.5	
	
No. of residues	1006	
	
No. of water molecules	358	
	
***B factor (Å***^2^**)**		
	
Protein atoms	64.6	
	
Water atoms	45.0	
	
***Stereochemistry***		
	
rmsd bond length (Å)	0.01	
	
rmsd bond angle (°)	1.6	
	
***Ramachandran Plot***		
	
Prefered regions (%)	66.9	
	
Allowed regions (%)	33.1	

^a ^Numbers in parentheses are values in the highest resolution shell.

^b ^R_merge _= ∑ | I_obs _- <I>|/∑ <I> summed over all observations and reflections

### Generation of plasmids and transgenic *P. falciparum *cell lines

Transgenic parasites expressing GFP chimeras were generated by cloning either the full length PfNapS coding region or truncated portions of it into the pARL vector [23], where expression of chimeric protein is under the influence of the *crt *promoter. Each DNA construct was confirmed by sequencing. 100 ug plasmid DNA (Qiagen) was transfected by electroporation into *P. falciparum *3D7 parasites which were cultured in the presence of 2 nM WR99210 [24]. To generate a PFI0930c (PfNapS gene) deletion construct 5' and 3' targeting sequences were amplified using the primer pairs aw771/772 and aw773/774 respectively. The amplified targeting sequences (613 bp and 530 bp) were cloned into pCC-1 vector [25] using SacII/SpeI for the 5' and EcoRI/AvrII for the 3' segment, resulting in the pCC-1ΔPfNapS plasmid.

(aw771: atcccgcggATCACATTGTAATTAAGC;

Aw772: gatactagtGAAAGGCATCAAAGGATCATC;

Aw773: atcgaattcGTGCCCATGAACAAATGAAC;

Aw774: gatcctaggCATCAAATTCTTCTAAGCC)

The plasmid DNA was proliferated in *Escherichia coli *PMC103 cells and purified with a Qiagen Maxit kit, before 80 ug were taken up in cytomix and electroporated into 3D7 *P. falciparum *infected human erythrocytes (O+) using standard procedures [24]. Parasites were cultured using standard conditions and parasites containing the plasmid were selected in the presence of 2 nM WR99210 [26]. The resulting parasite population was then subjected to two off/on drug cycles for 21 days each to encourage the loss of free plasmid. After each addition of WR99210, a subpopulation was selected on 231 nM 5-fluorocytosine in the presence of WR99210 (to encourage double recombination events) and analysed via Southern blot.

### SDS-PAGE and western blotting

Parasite cultures were synchronized by sorbitol treatment [27] and saponin pellets of trophozoite parasites were prepared. The parasite-derived proteins were fractionated on a 10% Bis-Tris precast gel (Invitrogen) using MOPS buffer. Proteins were electrophoretically transferred to a nitrocellulose membrane (0.22 um, Millipore) before western blot was performed using standard procedures. Antibodies used were mouse anti-GFP (1:1000, Roche) and horseradish peroxidase-coupled sheep anti-mouse IgG (1:2000, Chemicon, Melbourne, Australia). Protein bands on the western blots were visualized using a chemiluminescent substrate (ECL, GE).

### Microscopy

GFP-expressing infected erythrocytes were tightly synchronized using sorbitol and studied live at ambient temperature. Cells were viewed with a Plan-Neofluar 100× 1.3 oil objective on a Zeiss Axiovert 200 M Live Cell Imaging Inverted Microscope equipped with a AxioCam MRm camera and primarily processed with AxioVision 4.4 deconvolution software package. Captured images were then further processed using Photoshop and ImageJ software [28]. Pictures were adjusted to gain optimal contrast to visualize features of interest.

### Protein expression, purification, fluorescent labeling and ELISA-based binding studies

All histones (purchased from Roche diagnostics) were dissolved in autoclaved distilled water. PfNapS protein was expressed and purified using standard protocols described previously [5,6]. Site directed mutagenesis was carried out using QuickChange^® ^II Site-Directed Mutagenesis Kit. PfNapS was labelled with 6-acryloyl-2-dimethyl-aminonaphthalene (acrylodan) dye (from Invitrogen) using the protocol described by Prendergast *et al *[29] and Hibbs *et al *[30] with a few modifications. Acrylodan dye is an extrinsic fluorophore and its fluorescence emission is highly sensitive to its local microenvironment when bound to the protein. It exhibits changes in both intensity and emission wavelength that reflects the effective dielectric constant of the microenvironment around the fluorophore. PfNapS was labelled with the dye using the protocol described by Prendergast *et al *[29] and Hibbs *et al *[30]. Stock concentration of dye was prepared in dimethyl formamide. Purified protein was incubated with excess of dye in phosphate buffer (50 mM, pH = 7.5) overnight in the dark at 4°C. Unbound dye was removed using Superdex^® ^75 10/300 GL (GE healthcare). Binding of labelled PfNapS with H3 and linker histone H1 was studied using similar protocols described previously [6].

### Steady state fluorescence measurement

Steady state fluorescence measurement was taken on Perkin Elmer LS50B fluorescence spectrophotometer. The sample was titrated with histones H3, H4, H2A, H2B, histone tetramer { [(H3-H4)_2_]} and histone octamer (two heterodimers of H2A-H2B and a tetramer of H3-H4). Fluorescence of buffer was subtracted as control. Excitation was done at 360 nm and emission was scanned from 400 to 550 nm. Both the slits were 5 nm wide open and the scan speed was set to 100 nm per minute. All fluorescence emission data are calculated as mean of at least two replicate experiments.

## Results and Discussion

### Structure determination of PfNapS and structural comparisons with PfNapL, hSET, yNAP-1 and Vps75

**Figure 1:**
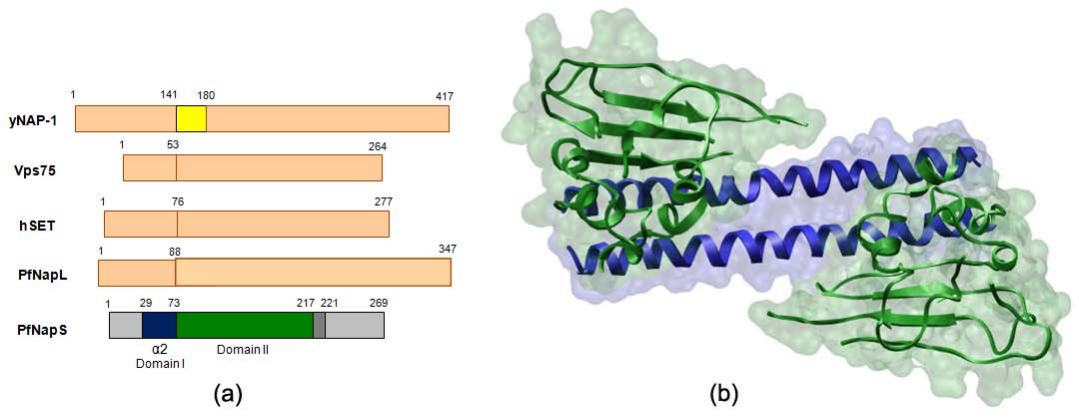
**Overall structure of PfNapS**. **(a) **Domain diagram for NAP/SET crystal structures determined so far. Architecture of PfNapS, yNAP-1, Vps75, hSET and PfNapL highlighting the 'accessory domain' in yNAP-1 (coloured yellow) which is missing in the remaining 4 structures (set to scale). **(b) **Structure of the PfNapS dimer. Each monomer of PfNapS contains domain I comprised of dimerization helix α2 and domain II comprised of a β sub-domain (4 anti-parallel β strands) and α-helices.

A construct of PfNapS consisting residues 29-221 (molecular weight ~23 kDa) crystallized in orthorhombic space group P2_1_2_1_2_1 _with solvent content of 55% (the asymmetric unit contains three dimers of PfNapS). Crystal structure of PfNapS was determined using selenium-SAD (single anomalous dispersion) technique at 3.2 Å and refined using native data to 2.8 Å resolution (Table 1). Most residues of PfNapS exhibited clear electron density. Four C-terminal residues (aa 218-221) were disordered as were regions 142-150 and 168-181. The final refined model of PfNapS has R_factor _and R_free _values of 28.2% and 31.45% respectively (Table 1). The domain architecture and the overall fold of PfNapS are similar to PfNapL, yNAP-1, hSET and Vps75 [7-12] (Figure 1a-b, 2a-c). Functional, cellular localization and sub-domain properties of these five structures are summarized in Table 2 and 3[6,7,31-33]. Each monomer of PfNapS contains domain I comprised of dimerization helix α2 (aa 33-73). The monomer also contains domain II, which is comprised of α-helices [α3 (aa 81-85), α4 (aa 99-102)] and a β sub-domain containing 4 anti-parallel β-strands (aa 106-158). The 4^th ^β-strand in PfNapS is composed of a random coil similar to hSET. Finally, there are two α-helices on the opposite side of the β sub-domain [α7 (aa 200-211 and α8 (aa 214-216)] (Figure 1b). It was observed that each of the six chains of PfNapS in crystallographic asymmetric unit differ mainly in the orientation of their dimerization helix α2 and the loop regions between the β-strands in the β sub-domain suggesting possible points of flexibility.

**Table 2:** Localization details of PfNapS, PfNapL, hSET, yNAP-1 and Vps75.

Protein, PDB code	Annotation	Localization	r.m.s.d of Cα, Å (number of Cα matched)	Length of dimerization helix α2 (r.m.s.d, Å)
**PfNapS, **3KYP	malaria parasite NapS	Primarily nuclear		41

PfNapL, 3FS3[6-8]	Malaria parasite NapL	Primarily cytoplasmic	1.94 (144)	51 (0.9)

hSET, 2E50[10,31]	Human SET/TAF-Iβ/INHAT functional domain	Nucleocytoplasmic	1.6 (139)	54 (0.75)

yNAP-1, 2AYU[9,32]	Yeast NAP-1	Nucleocytoplasmic	1.9 (112)	51 (1.0)

Vps75, 3CDM[11,12,33]	Yeast NAP	Primarily nuclear	2.0 (124)	42 (0.9)

Comparison of root mean square deviation (r.m.s.d) of NAP/SET monomers and length of dimerization helix α2 with PfNapS monomer.

**Table 3:** Comparison of pI (isoelectic point) of sequence stretches for all the 5 crystal structures

pI (isoelectric point)	Entire protein sequence	Protein sequence crystallised	Missing N-terminal	Missing C-terminal
**PfNapS**	4.4	5.4	5.2	3.0

PfNapL	5.0	7.0	4.9	3.3

hSET	4.2	4.9	-	2.6

yNAP-1	4.3	4.5	5.2	3.5

Vps75	4.9	5.2	3.4	3.4

**Figure 2:**
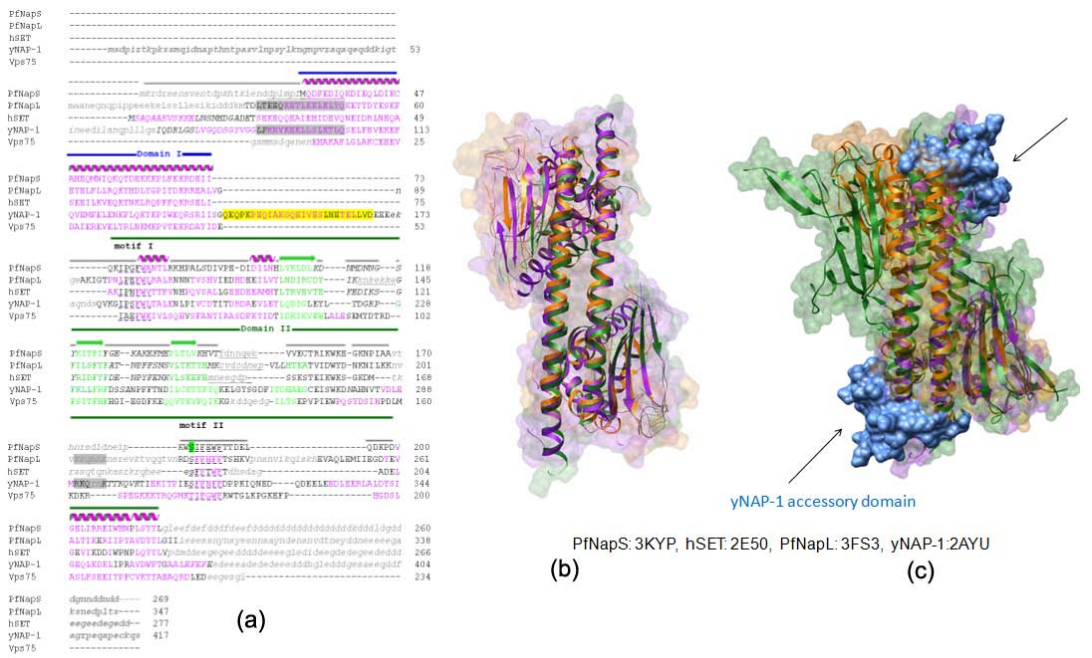
**Comparison of PfNapS with yNAP-1, Vps75, hSET and PfNapL (a)**. Structure-based sequence alignment of PfNapS, yNAP-1, Vps75, hSET and PfNapL. Residues constituting α-helices and β-sheets in all the individual structures are coloured magenta and green respectively. Domains I and II of PfNapS are indicated using blue and green bars respectively. Conserved hydrophobic motifs are dotted underlined in purple and indicated as 'motif I' and 'motif II'. The 'accessory domain' of yNAP-1 is foreground coloured yellow. The predicted NES and NLS motifs in yNAP-1 are shown in a shaded box coloured grey. The corresponding NES and NLS motifs in PfNapL are also shown in a shaded box coloured grey. Disordered/missing residues are in small caps and coloured grey. Residues that could not be aligned are shown in italics. **(b) **Superposition of PfNapS dimer structure (orange) onto hSET (PDB code: 2E50, green) and PfNapL (PDB code: 3FS3, purple). **(c) **Superposition of PfNapS dimer (coloured orange) onto hSET (PDB code: 2E50, green) and yNAP-1 (PDB code: 2AYU, purple). The 'accessory domain' of yNAP-1 which is absent in others is coloured blue.

The PfNapS dimer has a characteristic shape and fold previously seen for yNAP-1, Vps75, hSET and PfNapL dimers (Figures 1a-b, 2a-c) [7-12]. In PfNapS, 23 residues of the dimerization helix α2 and four residues of α7 and α8 (which lie in domain II) contribute to dimer formation via hydrogen bonding and hydrophobic interactions (Asp32, Phe33, Ile36, Gln37, Ile40, Leu43, Asp44, Lys45, Cys47, Glu50, Gln51, Ile54, Gln55, Gln57, Tyr58, Lys61, Lys62, Leu65, Phe66, Lys68, Arg69, Ile72, Ile73 and His210, Pro212, Leu313, Leu217 respectively). The overall sequence identity of PfNapS with yNAP-1, Vps75, hSET and PfNapL is 20, 17, 25 and 18% respectively. Similar to PfNapL and other NAPs in the malaria parasite [7], PfNapS is also evolutionary distant from homologs in yeast and human (Additional file 1). The length of the dimerization helix α2 varies in all the five proteins and PfNapS has the shortest such helix comprising of 41 residues (Table 2). The root mean square deviation (r.m.s.d) of PfNapS with yNAP-1, hSET, Vps75 and PfNapL is 1.9, 1.6, 2.0 and 1.9 Å respectively (Table 2; Figure 2a-c). PfNapS exhibits the highest sequence identity (25%) as well as structural similarity with hSET. Also, the r.m.s.d. of PfNapS is lowest with hSET at 1.6 Å indicating high structural identity (Table 2; Figure 2a, b). The N-terminal in PfNapS (aa 1-28) is missing in the present construct whereas in hSET the N-terminal is ordered (aa 1-15) and forms a helical structure α1 (aa 2-12). The region 195-200 of domain II in hSET is disordered and its corresponding region (aa 191-196) in PfNapS has weak electron density. The helices α7 and α8 on side of the β sub-domain of PfNapS are similar in length and orientation to hSET. Interestingly, the disorder in the present structure of PfNapS is also closest to hSET structure (Figure 2a, b).

The overall sequence identity of PfNapS with yNAP-1 and Vps75 is 20 and 17% respectively. The differences with respect to yNAP-1 and Vps75 are in the overall structural orientations of secondary structures (Table 2; Figure 2a, c). The major difference with yNAP-1 is absence of the 'accessory domain' in PfNapS. It has been earlier shown that this 'accessory domain' is absent in PfNapS, PfNapL and their homologs from other *Plasmodium *species [7]. A sequence comparison of PfNapS with other apicomplexan parasites like *Toxoplasma*, *Babesia *and *Theileria *revealed that this domain region is absent in them too (Additional file 2). Since the 'accessory domain' is absent in Vps75 and hSET as well (which are primarily localized in the nucleus), it remains unclear what role this domain plays in nuclear shuttling of these proteins, as was suggested for yNAP-1 [7-9]. The region 168-181 of PfNapS is disordered, however, Vps75 has an ordered helical content in this region (aa 153-176) and yNAP-1 has anti-parallel β-strands containing the nuclear localization signal (NLS). Also, comparisons with the identified NLS in yNAP-1 have revealed that PfNapS lacks an NLS which is also the case for Vps75.

A sequence alignment of PfNapS with other homologs from *Plasmodium *species (*Plasmodium vivax, Plasmodium berghei, Plasmodium knowlesi, Plasmodium yoelii *and *Plasmodium chabaudi*) revealed high sequence conservation amongst them (75-90%). Further analysis showed that the N-terminal exhibits lower conservation but the C-terminal contains acidic stretches in all of these proteins (Additional file 1). Dimer contributing residues of the dimerization helix α2 and domain II are well conserved in all structural homologs. Residues implicated in PfNapL for histone recognition are not conserved in PfNapS suggesting variable binding site for histones [7]. Also, a comparison of the residues from hSET mutagenesis data revealed that corresponding residues in PfNapS and its homologs from *Plasmodium *and other apicomplexans show overall weak conservation (Additional file 1).

### Gene knockout studies of PfNapS and its localization in the parasite

To assess the role of PfNapS *in vivo*, disruption of the corresponding gene was attempted from genome of *P. falciparum *3D7 strain. Despite several attempts, the gene could not be disrupted indicating an essential role for this gene (Additional file 3). This observation is consistent with unsuccessful attempts to disrupt NapS in the murine malaria parasite *P. berghei *[34]. Due to the current technological limitations imposed by the *Plasmodium *systems, the essentiality of PfNapS could not be formally proven; however, these results provide circumstantial evidence for a very important role played by PfNapS. Gene knockout phenotypes in several other studied organisms have indicated the essentiality of nucleosome assembly proteins. It has been shown that gene ablations in mouse and *Drosophila *cause embryonic lethality whereas in yeast cells exhibit growth defects [35-37]. Therefore, it is not surprising that the malaria parasite likely requires this gene for survival.

**Figure 3:**
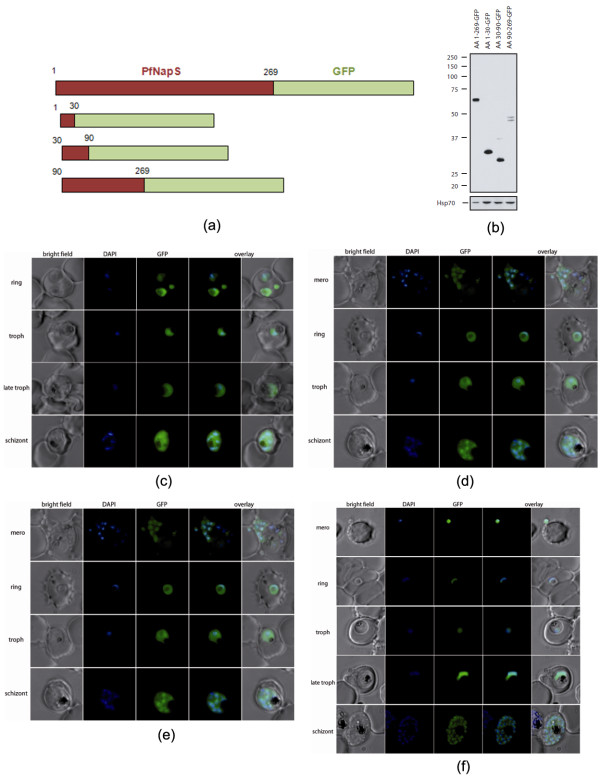
**Stage specific expression of PfNapS-GFP chimeras (a)**. Deletion constructs of PfNapS-GFP. **(b) **Western blot analysis of transgenic *P. falciparum *cell lines expressing GFP-tagged versions of PfNapS with antibodies against GFP. PfHsp-70 expression was detected as a loading control. Localization of **(c) **PfNapS-full length-GFP **(d) **aa1-30-GFP **(e) **aa30-90-GFP and **(f) **aa90-269-GFP in parasite merozoite, ring, trophozoite, late-trophozoite and schizont stages. The first column shows DIC (Differential Interference Contrast) images, followed by images with a nuclear DAPI stain, GFP fluorescence, an overlay of the nuclear and the GFP localization and overlay of all four images.

It has been earlier shown that PfNapS is expressed during all parasite blood stages, and immunofluorescence assays suggested a close association of PfNapS with parasite nucleus [5,6]. To address the issue of potential sequences within PfNapS which might target this protein to the parasite nucleus (in the absence of a clear NLS motif), full-length PfNapS was fused with green fluorescence protein (GFP) and these gene fusions were expressed episomally in wild-type parasite. The protein chimeras showed a nuclear localization in all stages of erythrocytic life-cycle as determined by co-localization with the nuclear stain DAPI (4', 6-diamidino-2-phenylindole; nuclear stain) (Figure 3a, c-f). Western blot analysis using anti-GFP antibodies confirmed the expression of fusion proteins (Figure 3b). All fused proteins ran at the expected sizes except for residues 30-90-GFP which resulted in a band at around 29 kDa rather than the predicted 34 kDa. One possible explanation might be the relative high content of glutamines and glutamic acids in this fusion protein resulting in aberrant protein mobility. The three truncated forms consisting of aa1-30-GFP, aa30-90-GFP and aa90-269-GFP (Figure 3a) showed different localizations in live cell fluorescence microscopy: aa90-269 showed a similar nuclear localization to the full-length chimera, whereas aa1-30-GFP and aa30-90-GFP showed fluorescence in the parasite cytoplasm. Therefore, it seems likely that the region responsible for targeting PfNapS to the nucleus is contained within PfNapS residues 90-269. The localization of these fusion proteins did not alter during the parasite life-cycle stages in immunofluorescence assays (Additional file 3). In the well-studied yeast NAP (yNAP-1), residues 290-295 ('RKQRNK') have been experimentally identified as an NLS [9]. It has been previously shown that the corresponding region in PfNapL (aa 203-207, 'KKQHNK') is disordered in the crystal structure, and in any case PfNapL seems resident in the parasite cytoplasm [7] (Table 2; Figure 2a). In the case of nuclear associated PfNapS, the corresponding region is again disordered (aa 172 to 177-'NRSDLD') [7,9] (Table 2, Figure 2a-b). Therefore, GFP-fusion transfection data suggest that PfNapS likely contains NLS-like motif in residues 90-269 but the set or sets of motifs, which direct PfNapS to the nucleus remain to be discovered.

### PfNapS-histone interaction using fluorescence spectroscopy

**Figure 4:**
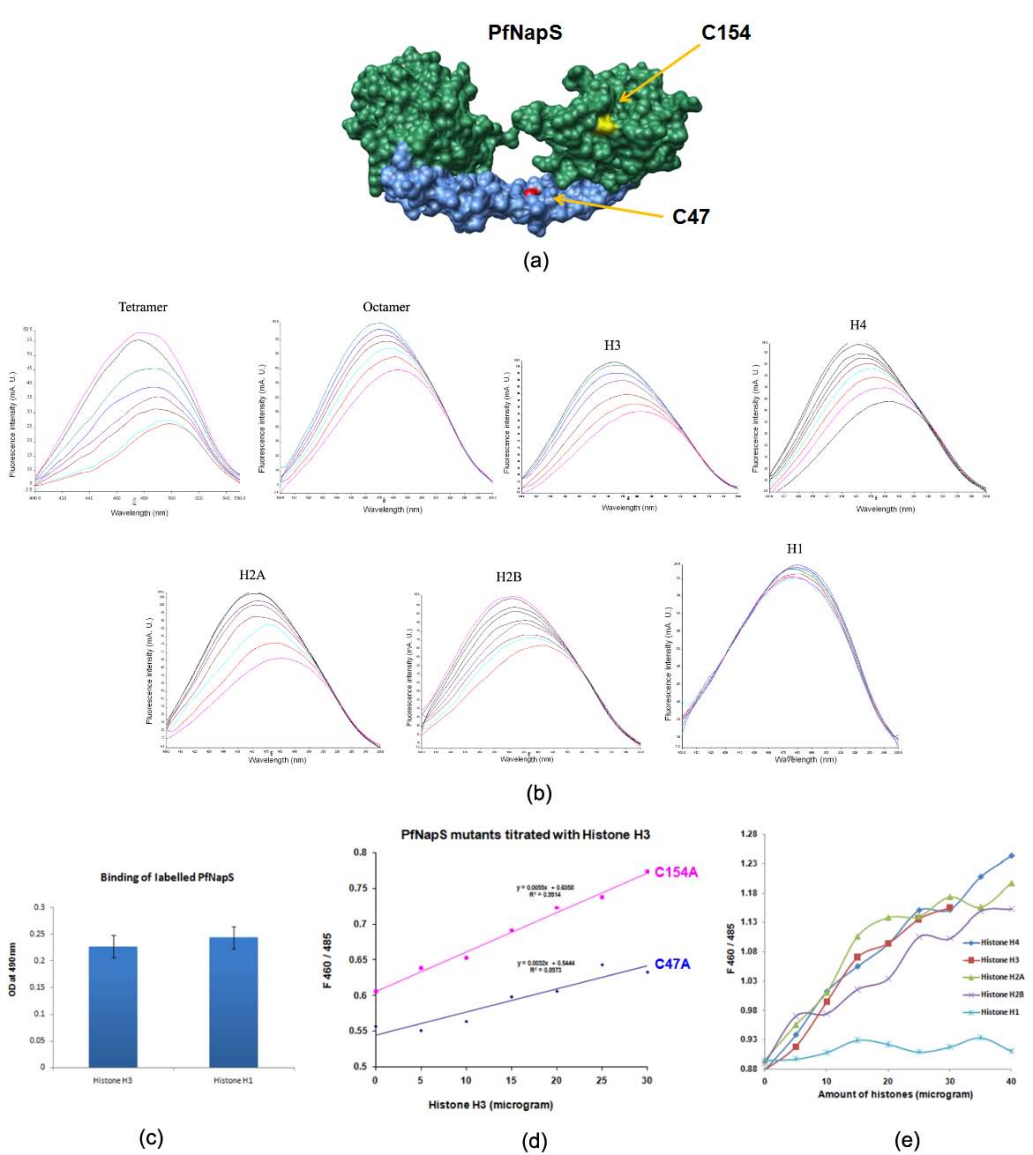
**PfNapS-histone interaction studies using fluorescence spectroscopy (a)**. PfNapS dimer is shown as molecular surface. Domains I and II are coloured blue and green respectively. The residues Cys47 and Cys154 are coloured red and yellow and are indicated. **(b) **Fluorescence emission spectra of PfNapS in presence of increasingconcentrations of histones. A hypsochromic shift (blue shift) was observed onbinding of histones. However in case of histone H1 no such change wasobserved. Fluorescence intensity was measured after incubating75 micro gram of PfNapS protein with 5, 10, 15, 20, 25, 30, 35, 40, 45, and 50 micrograms ofhistones. All experiments were repeated thrice. **(c) **Effect of labeling on binding of PfNapS to histones H3 and H1. **(d) **Graph representing hypsochromic shift (F460/485) in fluorescence of two PfNapS mutants as a function of amount of H3 histone. **(e) **Graph representing differential binding of linker histone H1 with respect to core histones. There is no shift in fluorescence on binding of H1, however, core histones show hypsochromic shift (F460/485) in fluorescence.

Structural analysis reveals two free surface exposed cysteine residues (Cys47 and Cys154) in PfNapS dimer. The first residue Cys47 is present at concave region of the dimerization helix α2 whereas Cys154 is present on outer surface of domain II (Figure 4a). These cysteines were labelled on PfNapS using molecular environment sensitive dye 6-acryloyl-2-dimethyl-aminonaphthalene (acrylodan) which binds covalently to surface exposed cysteine residues. Post modification, the tagged protein (PfNapS^dye^) was passed through gel filtration column to remove unbound dye. PfNapS^dye ^was then used in histone interaction studies. Upon titrating PfNapS^dye ^with histone monomers and histone oligomers a gradual blue shift was observed along with an increase in the quantum yield (Figure 4b). This chromic shift reaches a saturation level and then no further blue shift or increase is observed upon addition of either the same histone or a different one. These data suggest embedding of the tagged cysteines upon binding of PfNapS^dye ^to histones. Such interaction likely causes transition of dye from a polar (fully exposed to solvent) to a non-polar environment (i.e. burial at Nap-histone interface).

To further investigate probable histone binding region(s) on PfNapS, these two surface cysteine residues on PfNapS were mutated independently (mutants are referred to as C47A and C154A). On plotting the change in fluorescence as a function of histone concentration, it was observed that in the absence of histone, the chromic shift was similar for these mutants. This analysis suggests that both the cysteine residues experienced same solvent environment. Upon adding histones, a blue shift was observed along with an increase in the quantum yield suggesting that C47A and C154A might play a role in histone binding. However, a higher change in the chromic shift in C154A (about two times) in comparison to C47A (Figure 4d) possibly suggests a bigger role of C154A in the binding interaction.

Interestingly, no chromic shift or increase in the quantum yield in fluorescence was observed on titrating the PfNapS^dye ^with linker histone H1 indicating no change in the dielectric constant of the microenvironment around the fluorophore (Figure 4e). These data suggest a different mode and/or region of binding of linker histone H1 to PfNapS as opposed to the core histones. To rule out the possibility that the dye could be affecting binding of histone H1 to PfNapS, interaction of labelled PfNapS with histone H1 was analysed and compared it with binding with histone H3. No significant difference was observed in binding of these histones to PfNapS (Figure 4c). These data are in agreement with previously published work, which suggested differential binding of NAPs to linker histone H1 when compared with core histones and histone oligomers [38,39]. Interestingly, no clear homolog for histone H1 has been identified in *Plasmodium *or indeed in any apicomplexan organism so far. However, histone H1 sequences tend to vary and it is likely that their overall structure is more conserved. It would be very unusual if apicomplexans lacked linker histone H1 altogether. The above experiments were done with all canonical histones with the assumption that apicomplexans will have a structural homolog of linker histone H1.

### Comparison of histone binding characteristics of NAP/SET structures

Mutagenesis studies performed previously on hSET had revealed important residues in domain II that affect the binding of hSET to both core histones and dsDNA [10]. These residues were mapped onto the PfNapS dimer and most of the corresponding ones are not conserved with hSET except for Lys163 and Thr190 (Table 4). Only six of the 12 residues that affect binding of hSET to histones (completely or to a marginal extent) are conserved in PfNapS. In the previous study on the cytoplasmically resident PfNapL, six residues of PfNapL were mutated based upon the hSET mutagenesis data and it was shown that these six had no effect on the binding of PfNapL to histones [7]. These six residues are mostly conserved amongst PfNapL and PfNapS with only the residues His227 and Tyr259 of PfNapL replaced in PfNapS by glutamine and proline residues respectively (Table 5). Three residues of PfNapL have been earlier identified that potentially contribute to histone recognition, based on Asf1-histone complex analysis [7,40,41]. Although two of these residues of PfNapL are not conserved in PfNapS, the third is in fact an alanine in PfNapL and a cysteine (Cys154) in PfNapS. Interestingly, new fluorescence data indicates a role for region encompassing this cysteine (Cys154) in interaction with all core histones, except with linker histone H1. In summary, the studies on PfNapL and PfNapS together suggest a region or regions in domain II of nucleosome assembly proteins which likely contribute to histone recognition.

**Table 4:** Structure-based comparison with hSET mutagenesis data of residues which effect binding (completely or partially) to histones

	hSET	PfNapL	PfNapS	Vps75	yNAP-1
1	S162	D192	E161	Q150	D278

2*	K164	K194	**K163**	D152	H281

3	D165	N195	N164	S153	N282

4	T191	H227	E187	G181	N314

5*	T194	T230	**T190**	R184	D317

6	D195 (disordered)	S231	T191	W185	D317

7	D202	Y259	P198	D198	Y342

8*	E203	E260	**D199**	S199	S343

9*	E206	L263	**E202**	S202	E246

10*	K209	K266	**R205**	S205	K349

11	D210	E267	R206	E206	D350

12*	D211	R268	**E207**	E207	K351

13	E187 (disordered)	D223	W183	K177	E320

*conserved in PfNapS

**Table 5:** Structure-based comparison with PfNapL mutagenesis data

	PfNapL	PfNapS	Vps75	yNAP-1	hSET
**Effect binding**					

1	**I136**	N112	S95	-	F115

2	**I147**	K120	S104	K230	R123

**No effect on binding to PfNapL**		**PfNapS**	**Vps75**	**yNAP-1**	**hSET**

1*	**D192**	E161	Q150	D278	S162

2	**H227**	E187	G181	N314	T191

3*	**T230**	T191	R184	D317	T194

4	**Y259**	P198	D198	Y342	D202

5*	**E260**	D199	S199	S343	E203

6*	**K266**	R205	S205	K349	K209

7	**D223**	W183	K177	E320	Disordered

*conserved in PfNapS

### Phosphorylation status

**Figure 5:**
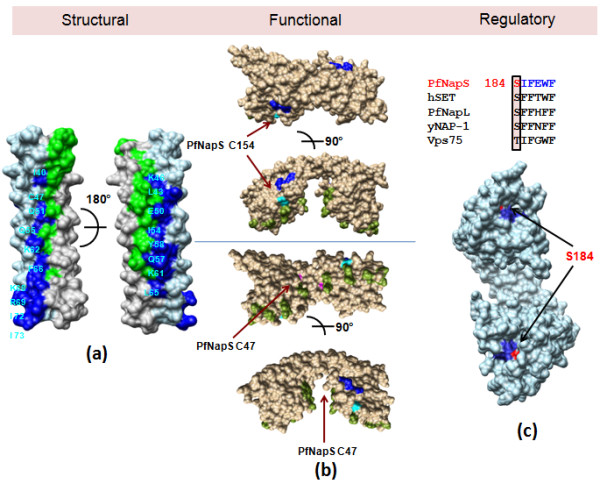
**Overall structural, functional and regulatory comparisons between NAP/SET proteins based upon 5 crystal structures **[7-12]. **(a)**. PfNapS dimerization helix α2 is shown as molecular surface. Conserved dimer contributing residues between all 5 crystal structures are mapped onto PfNapS. Residues from chains A and B are coloured blue and green respectively and residues from chain A are labelled. **(b) **hSET is shown as molecular surface and coloured brown. Residues from hSET mutagenesis analysis are coloured green and corresponding residues from PfNapL mutagenesis are coloured blue. The corresponding residues for the two cysteines from PfNapS fluorescence data are coloured cyan and are indicated with arrows. **(c) **PfNapS dimer is shown as molecular surface and coloured sky blue and the conserved hydrophobic motif is coloured blue. Surface exposed serine residue is coloured red.

It has been earlier shown that both PfNapL and PfNapS are phosphorylated by casein kinase II (CKII) [5,6]. Programs NetPhos and NetPhosK predicted phosphorylation sites on PfNapS of which three residues Ser91, Thr190 and Thr191 (CKII phosphorylation site) are surface exposed in the structure. All these residues have corresponding serine and/or threonine residues respectively in hSET and PfNapL, which are also surface exposed. However, the site Ser91 of PfNapS is replaced by cysteine and alanine residues and Thr190 is replaced by aspartate and arginine residues in yNAP-1 and Vps75 respectively. Thus, the phosphorylation sites predicted for PfNapS are conserved in PfNapL and in hSET but not in yNAP-1 and Vps75. Further, the three phosphorylation sites which have been earlier identified for yNAP-1 lie in the 'accessory domain' region which is absent in PfNapS, PfNapL, hSET and Vps75. Interestingly, the corresponding residues for Ser184 of PfNapS that lie in the conserved hydrophobic motif (SIFEWF) are conserved and surface exposed in all the five structures and could possibly represent a common phosphorylation site for NAP/SET proteins (Figures 2a, 5c). This conserved residue is not a predicted CKII site on any of these five proteins but nonetheless may be of significance given its conservation in these proteins.

In summary, these comparative structural analyses show that PfNapS, PfNapL, hSET, yNAP-1 and Vps75 contain conserved residues in the dimerization helix which contribute to dimer formation in all these five proteins (Table 6 and Figure 5a). Functional data on PfNapS indicate the inability to delete PfNapS gene suggesting its essentiality in the parasite. Transfection studies identify parasite nucleus as the site for localization of PfNapS. Fluorescence data analysis highlights two regions on PfNapS dimer which are likely to contribute to histone recognition (Figure 5b). Finally, it is likely that the region for histone recognition on NAPs and SET domains may be the outer face of domain II and base of the cavity in the underside of each dimer (Figure 5b).

**Table 6:** Conserved residues in PfNapS, PfNapL, yNAP-1, hSET and Vps75 which contribute to dimer formation

	Conserved in all 5 proteins	Conserved in 4 of 5	Conserved in 3 of 5
**PfNapS residues**	I40, L43, K46, C47, E50, Q51, I54, Q57, Y58, K61, K62, L65, K68, R69, I73, I73	D32, F33, I36, D44, Q55, F66	Q37

Yeast two-hybrid studies on interacting partners of PfNapS suggest a crucial role for this protein in numerous protein networks [42]. A total of 27 proteins have been shown to interact with PfNapS [42]. However, in the absence of independent experimental validation of each of these protein-protein interactions, it is premature to speculate on their biological relevance. Nonetheless, an attempt was made to classify these binding partners for PfNapS. Analysis suggests that three of the 27 proteins are predicted to be localized to the parasite nucleus (Additional file 4). Further, a total of six of 27 proteins are likely to be involved in chromatin modelling (Additional file 4). The crystal structures of these interacting proteins are unknown and, therefore, their binding modes with PfNapS remain unexplored. Clearly, these studies presented here pave the way for deeper dissection of numerous protein-proteins interactions that occur with PfNapS.

## Conclusions

The on-going structural, biochemical, gene knockout and localization studies on the two nucleosome assembly proteins from *P. falciparum *have revealed similar three-dimensional structures for the two proteins whilst having different localizations within the parasite. Based on extensive experimental analysis on both malaria parasite nucleosome assembly proteins, it is suggested that this critical pair of proteins provide a unique opportunity for the exploration as anti-malarial targets.

## Protein Data Bank Accession Code

The coordinates have been deposited in the public databank with PDB code 3KYP.

## Competing interests

The authors declare that they have no competing interests.

## Authors' contributions

JG solved the structure of PfNapS and along with AS analysed all data and wrote the manuscript. AK purified and crystallized seleno-met PfNapS and also performed the binding studies. MY, HB, SKJ assisted with the work presented. MR, MB and AGM performed the gene KO and transfection experiments. All authors assisted with manuscript preparation and read and approved the final manuscript.

## Supplementary Material

Additional file 1 **Phylogenetic tree of NAPs from various species showing greater evolutionary distance of malaria parasite NAPs from homologs in yeast and man (indicated by red arrows)**. Click here for file

Additional file 2 **Structure-based sequence alignment of PfNapS with its homologs from *Plasmodium *and other apicomplexans**. Residues that are identical and conserved within PfNapS and its homologs are colored red and green respectively. The histone binding residues from hSET and PfNapL mutagenesis experiments are shown in shaded box colored cyan and green respectively [7,10]. Click here for file

Additional file 3 **PfNapS gene knockout analysis**. Click here for file

Additional file 4 **Protein interacting partners of PfNapS based upon yeast two-hybrid data are shown which need further experimental validation**. Proteins are marked based upon their predicted localization - nuclear or cytoplasmic wherever possible. Click here for file
