# Supplementary material for: Structure, localization and histone binding properties of nuclear-associated nucleosome assembly protein from Plasmodium falciparum
Source: Malar J. 2010 Apr 8;9:90. doi: 10.1186/1475-2875-9-90 (PMC2873526; doi:10.1186/1475-2875-9-90)

Additional file 1: Phylogenetic tree of NAPs from various species showing greater evolutionary distance of malaria parasite NAPs from homologs in yeast and man (indicated by red arrows).

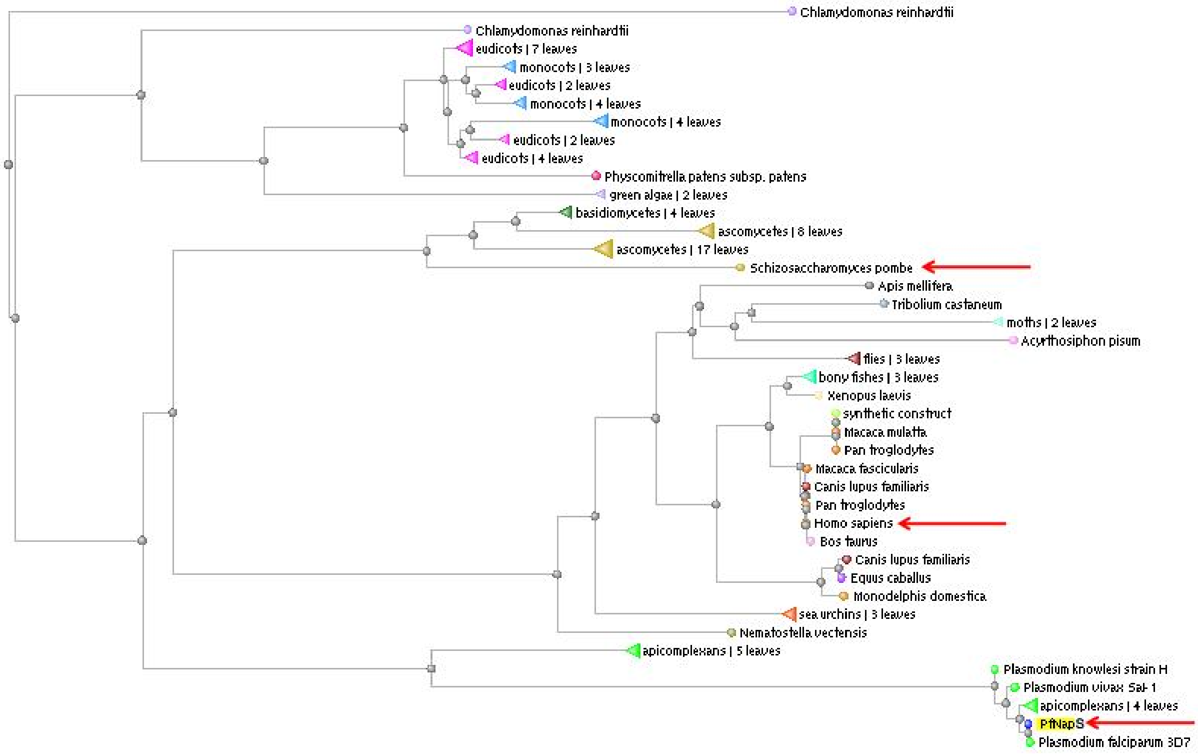

Supplement: Additional file 1 — Phylogenetic tree of NAPs from various species showing greater evolutionary distance of malaria parasite NAPs from homologs in yeast and man (indicated by red arrows). [file 1475-2875-9-90-S1.PDF]
