# Supplementary figures and images for: Structure, localization and histone binding properties of nuclear-associated nucleosome assembly protein from Plasmodium falciparum
Source: Malar J. 2010 Apr 8;9:90. doi: 10.1186/1475-2875-9-90 (PMC2873526; doi:10.1186/1475-2875-9-90)

**Additional file 3:** PfNapS gene knockout analysis

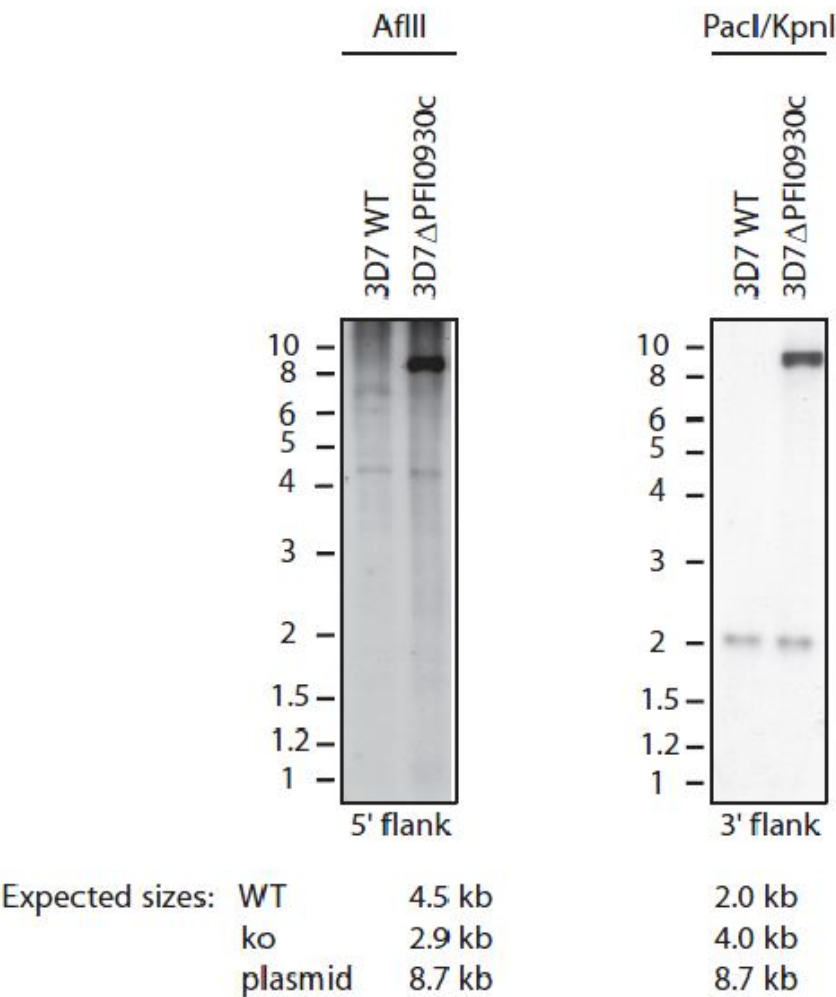

Supplement: Additional file 3 — PfNapS gene knockout analysis. [file 1475-2875-9-90-S3.PDF]
