# Supplementary material for: Structure, localization and histone binding properties of nuclear-associated nucleosome assembly protein from Plasmodium falciparum
Source: Malar J. 2010 Apr 8;9:90. doi: 10.1186/1475-2875-9-90 (PMC2873526; doi:10.1186/1475-2875-9-90)

Additional file 4: Protein interacting partners of PfNapS based upon yeast two-hybrid data are shown which need further experimental validation. Proteins are marked based upon their predicted localization – nuclear or cytoplasmic wherever possible.

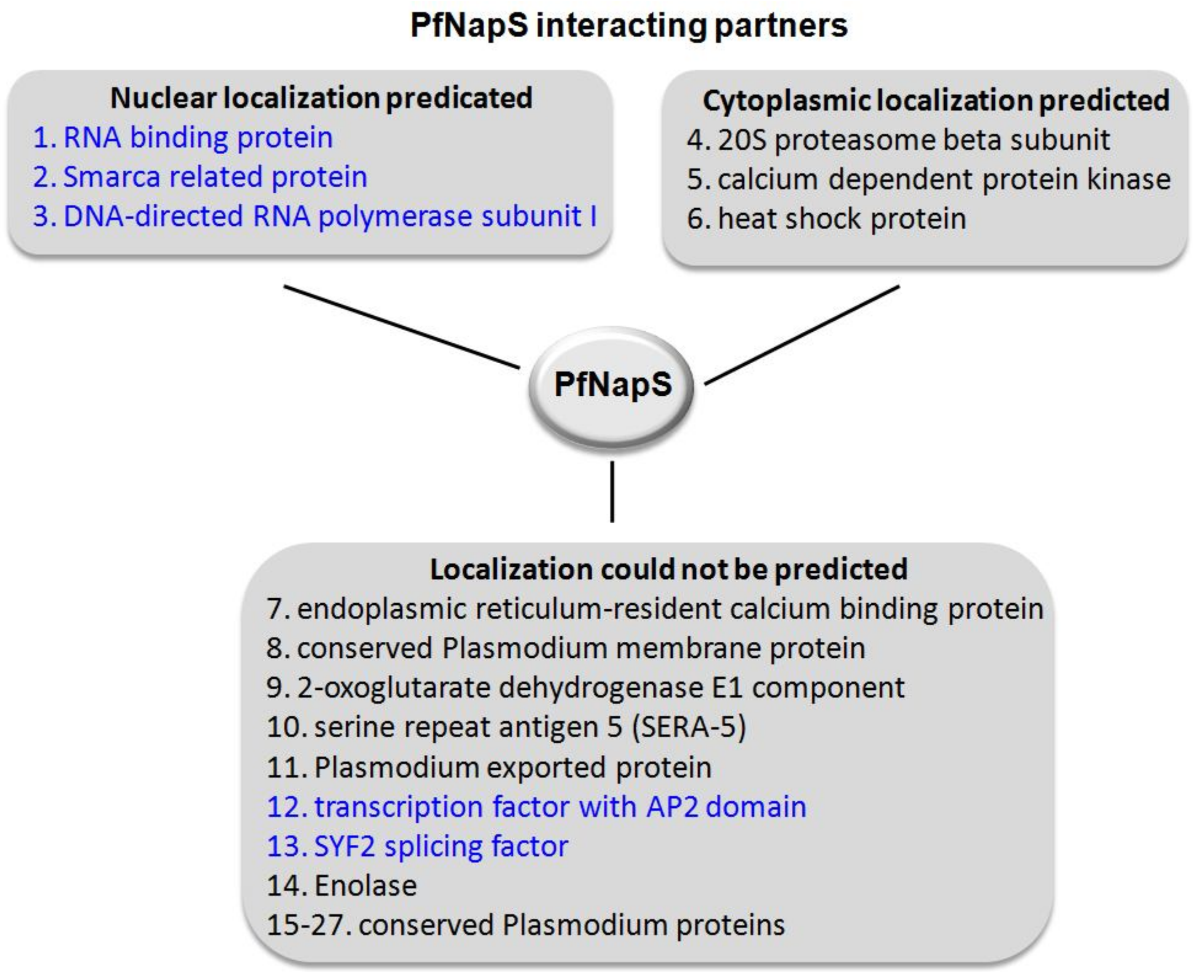

Supplement: Additional file 4 — Protein interacting partners of PfNapS based upon yeast two-hybrid data are shown which need further experimental validation. Proteins are marked based upon their predicted localization - nuclear or cytoplasmic wherever possible. [file 1475-2875-9-90-S4.PDF]
